# The microbiota in the intestinal and respiratory tracts of naked mole-rats revealed by high-throughput sequencing

**DOI:** 10.1186/s12866-018-1226-4

**Published:** 2018-08-22

**Authors:** Wei Cong, Jin Xing, Yufang Feng, Ji Wang, Rui Fu, Bingfei Yue, Zhengming He, Lifang Lin, Wenjing Yang, Jishuai Cheng, Wei Sun, Shufang Cui

**Affiliations:** 1 Laboratory Animal Centre, Second Military Medical University, No.8 Rd. Panshan, Yangpu District, Shanghai, China; 2 National Institutes for Food and Drug Control, Institute for Laboratory Animal Resources, No.31 Rd. Huatuo, Daxing District, Beijing, China

**Keywords:** Naked mole-rats, Microbiota, Cecum, Trachea, Bacterial diversity

## Abstract

**Background:**

The naked mole-rat (NMR, *Heterocephalus glaber*) is being bred as a novel laboratory animal due to its unique biological characteristics, including longevity, cancer resistance, hypoxia tolerance, and pain insensitivity. It is expected that differences exist between the microbiota of wild NMRs and that of NMRs in an artificial environment. Overall, the effect of environment on changes in the NMR microbiota remains unknown. In an attempt to understand the microbiota composition of NMRs in captivity, variability in the microbiota of the intestinal and respiratory tracts of two groups of NMRs was assessed under two conditions.

**Results:**

The results obtained by high-throughput sequencing revealed significant differences at the phylum, class, order, family and genus levels in the microbiota between the two groups of NMRs examined (first group in conventional environment, second group in barrier environment). For the trachea, 24 phyla and 533 genera and 26 phyla and 733 genera were identified for the first and second groups of animals. Regarding the cecum, 23 phyla and 385 genera and 25 phyla and 110 genera were identified in the microbiota of first and second groups of animals. There were no obvious differences between females and males or young and adult animals.

**Conclusions:**

Our results suggest that the intestinal and respiratory tract NMR microbiota changed during captivity, which may be related to the transition to the breeding environment. Such changes in the microbiota of NMRs may have an effect on the original characteristics, which may be the direction of further research studies.

## Background

Naked mole-rats (NMRs, *Heterocephalus glaber*) are a unique species of rodents exhibiting high longevity, tumor resistance, hypoxia tolerance, and pain insensitivity [1–4]. Due to these attributes, NMRs are widely used as mammalian models in biomedical research studies investigating aging, cancer, and neurobiology as well as other topics. As a novel laboratory animal, the NMR is strongly differs from previously used experimental animals. However, as wild NMRs live in underground caves, which is a relatively low-oxygen and dark environment, a difference may exist between the microbiota of wild NMRs and those that live aboveground.

High-throughput sequencing has become an important method for studying the structure, diversity and pathogenicity of the bacterial flora of humans and animals [5–7]. Overall, the microbiota plays a significant role in maintaining the health and well-being of the mammalian host and also assists in nutrient metabolism [8, 9] and affects the body’s immune function [10–12]. The microbiota is closely associated with the occurrence of tumors [13, 14], and the intestinal tract microbiota even exhibits antitumor immune effects [15]. As stated above, the NMR is characterized by longevity and anti-aging and cancer resistance. In an attempt to understand the composition of the NMR microbiota and its potential association with these properties, Debebe and colleagues [16, 17] preliminarily studied cultured bacteria from the bowels of wild NMRs by separating the gut flora and characterized the intestinal microbial ecosystem using next-generation sequencing. However, details regarding the microbiota of the intestinal and respiratory tracts of NMRs in captivity remain unknown. In this study, we sought to analyze the microbiota in the intestinal and respiratory tracts of NMRs in captivity in an effort to reveal the bacteria-related factors underlying the longevity of NMRs.

## Results

### Sequencing results and acquisition of operational taxonomic units (OTUs)

We assessed tracheal secretions and cecum contents of two groups of NMRs (*n* = 33 for the first; *n* = 30 for the second) using high-throughput sequencing. The NMRs studied were derived from one closed colony obtained from Second Military Medical University [18]. The first group was bred in a conventional environment (fed without strict control and housed in an unsterilized cage); the second group was bred in a barrier environment (fed with strict control and caged with sterilization before use).

Approximately 1.5 million filtered sequences were obtained for tracheal secretions (approximately 580,000 for the first group and approximately 930,000 for the second group) and approximately 2 million filtered sequences for cecum contents (approximately 690,000 for the first group and approximately 1.03 million for the second group). After removing chimeric sequences and non-target regions, the number of residual sequences was, on average, 20,571 reads per sample.

All sequences were classified for OTU analysis according to the similarity threshold setting (0.97). The tracheal results for the first group showed 24,115 OTUs, with an average of 2445 in each animal and an average of 421-bp high-quality sequences (the shortest sequence was 200 bp; the longest sequence was 451 bp). For the second group, 5470 OTUs, with an average of 396 bp (the shortest was 200 bp; the longest was 462 bp) were acquired by sequencing.

The number of OTUs in the first and second groups fit a normal distribution, and the difference in the number of OTUs was statistically significant (*P* < 0.001). For the first group, there was no significant difference in the number of OTUs (*P* = 0.224) between male and female NMRs; similarly, no significant difference in the number of OTUs (*P* = 0.074) between young (≤ 20 weeks) and adult (> 20 weeks) NMRs in was observed. Although the number of OTUs was not significantly different (*P* = 0.263) between male and female animals in the second group, there was a significant difference (*P* = 0.009) between young and adult animals.

Sequencing of the cecal contents in the first group resulted in 110,815 OTUs, with an average of 3358 OTUs per animal and an average read length of 416 bp (the shortest was 200 bp; the longest was 447 bp). A total of 9118 OTUs were sequenced for the second group of 30 NMRs, with an average read length of 385 bp (the shortest was 201 bp; the longest was 462 bp).

OTUs for the cecal contents were normally distributed and showed a significant difference (*P* < 0.001) between the first and second groups. There was no significant difference in the number of cecal OTUs (*P* = 0.479) between male and female NMRs and no significant difference in the number of cecal OTUs (*P* = 0.499) between the two age groups in the first group. In addition, the number of cecal OTUs was not significantly different (*P* = 0.263) between male and female animals or between young and adult animals (*P* = 0.685) in the second group.

**Fig. 1:**
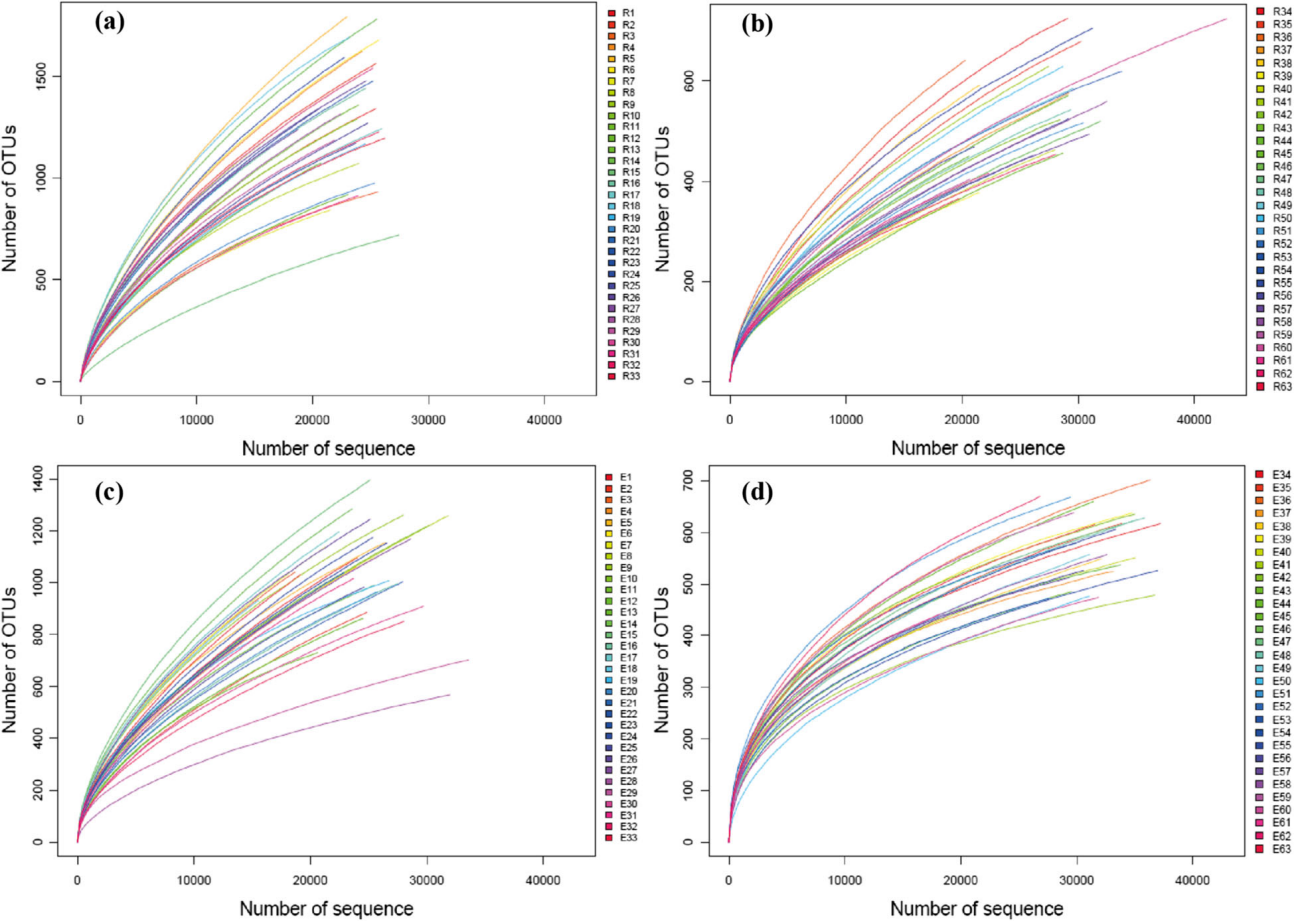
Rarefaction analysis of the different samples. **a**. Rarefaction curves comparing the number of reads with the number of OTUs based on DNA from the contents of (**a**) the trachea in the first 33 NMRs, **b** the trachea in the second 30 NMRs, **c** the cecum in the first 33 NMRs, and **d** the cecum in the second 30 NMRs

The sequencing data were analyzed by alpha diversity analysis, including the coverage of each sample library, Shannon index, Chao1 index, ACE index, and Simpson index (Table 1), and MOTHUR was used for rarefaction curve analysis (Fig. 1a, b, c, d). According to the rarefaction curves, there was no significant change in the number of OTUs, and the number of sequences was able to reflect the diversity in the ileocecum microbiota.

**Table 1:** Number of sequences retrieved, number of OTUs and alpha diversity indexes

Variable (Average)	Trachea		Cecum	
	1st collection	2nd collection	1st collection	2nd collection
Sequence number	17,560 ± 1786	30,872 ± 6892	20,571 ± 2280	34,470 ± 4870
OTUs	2445 ± 790	304 ± 74	3358 ± 887	304 ± 48
Coverage	0.915 ± 0.028	0.995 ± 0.001	0.893 ± 0.037	0.997 ± 0.001
Shannon	4.85 ± 0.77	2.67 ± 0.19	5.61 ± 0.73	3.17 ± 0.48
Chao1	5386.31 ± 1761.64	610.22 ± 141.98	8830.93 ± 2567.26	592.40 ± 122.48
ACE	8259.34 ± 2864.72	918.44 ± 238.74	13,870.80 ± 4780.53	674.65 ± 151.16
Simpson	0.075 ± 0.070	0.136 ± 0.026	0.034 ± 0.031	0.146 ± 0.088

### LEfSe (linear discriminant analysis effect size) analysis

**Fig. 2:**
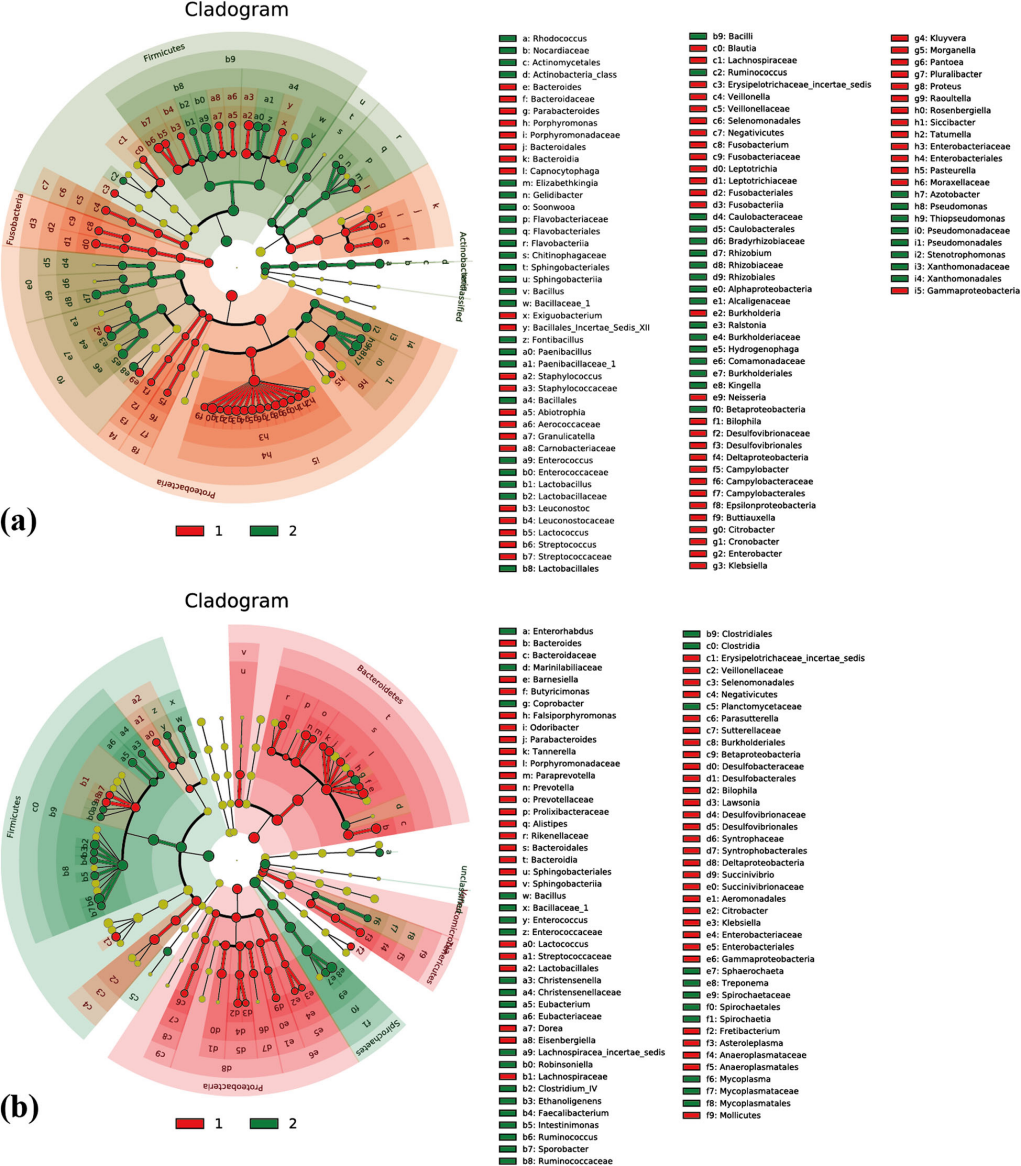
Cladogram of all 63 NMRs via LEfSe analysis. **a** Trachea; **b** cecum. Different colors represent different groups, and different color nodes in the branches represent the microbiota that plays an important role in the group. The yellow nodes represent a group of microbes that do not play an important role

In LEfSe analysis, differences between the two groups of NMRs indicated significant differences in the microbiota at the phylum, class, order, family and genus levels. For the trachea and cecum microbiota, 117 (65 + 52) and 93 (59 + 34) differentially abundant taxonomic clades, respectively, were obtained for the two groups of animals. LDA (linear discriminant analysis) scores were all higher than 2.4 (data not shown). The most significantly different taxa in the trachea were Enterobacteriaceae (Proteobacteria) in the first group and Pseudomonadaceae (Proteobacteria) in the second group. *Burkholderia*, *Neisseria*, *Pasteurella*, *Staphylococcus* and *Streptococcus* were clearly present in the trachea of the first group of animals but were effectively eliminated in the second group (Fig. 2a). Regarding the cecum microbiota, the greatest differences were exhibited by Bacteroidales (Bacteroidetes) in the first groups and Ruminococcaceae (Firmicutes) in the second. It was clear that Bacteroidetes and Proteobacteria accounted for a significant portion in the first group of animals, whereas *Citrobacter* and *Klebsiella* were controlled in the second group. *Bacillus* (Firmicutes) and *Spirochaetia* (Spirochaetes) were identified according to their characteristics (Fig. 2b).

### Comparison of microbiota at the phylum level

Overall, the results of microbiota analysis at the phylum level for the first groups of endotracheal samples demonstrated that of the 24 phyla identified, the content of Proteobacteria (66.90%) was the highest, followed by Firmicutes (18.32%), Bacteroidetes (10.10%), Fusobacteria (2.73%), unclassified (1.84%), Spirochaetes (0.06%) and Planctomycetes (0.01%). In the second group of animals, the microbiota comprised 26 phyla, with the highest levels for Proteobacteria (47.33%) and Firmicutes (41.91%), which accounted for nearly 90% of the population, followed by Bacteroidetes (2.97%), Actinobacteria (2.43%), unclassified (3.51%), Fusobacteria (0.18%) and Spirochaetes (0.17%).

**Fig. 3:**
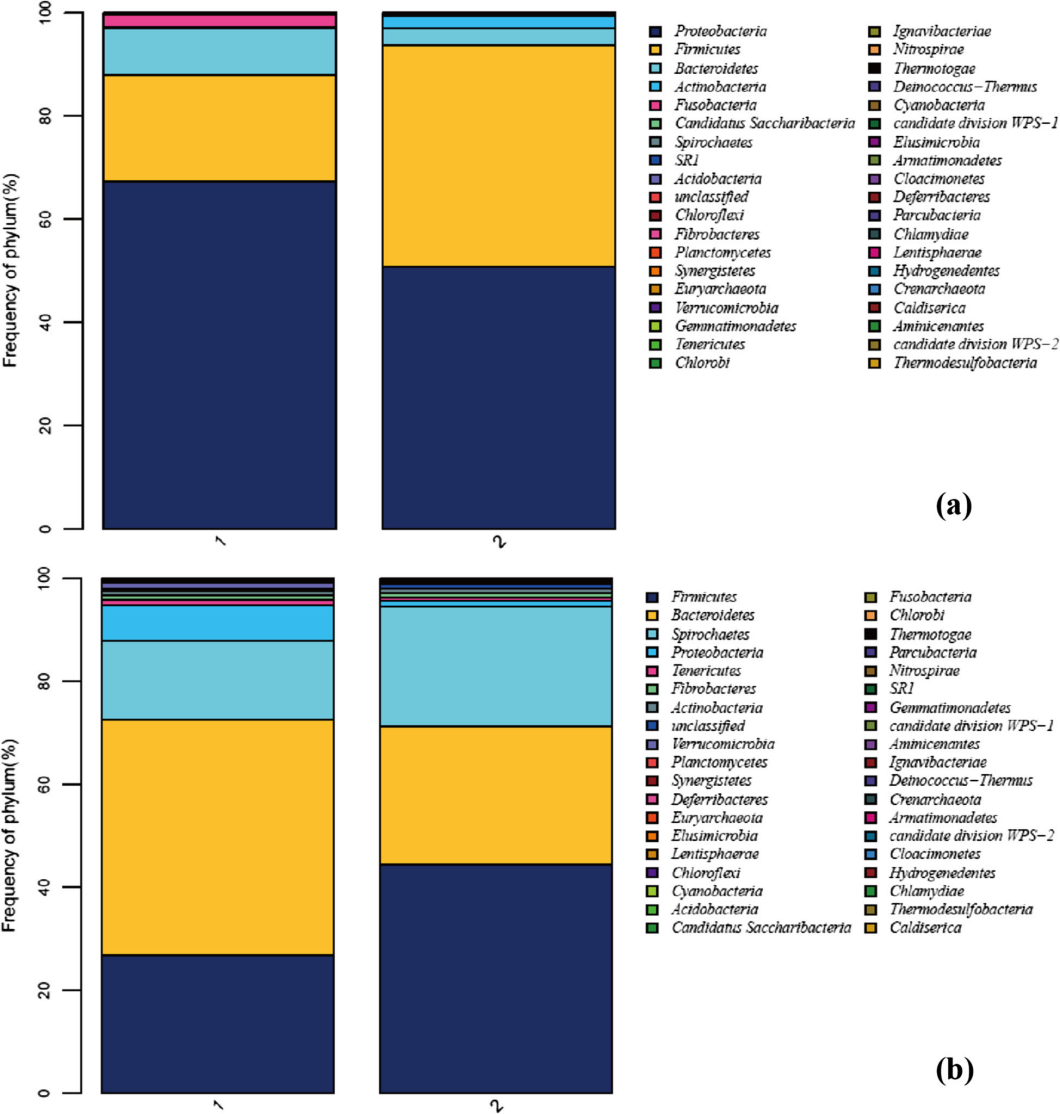
Overall distribution of the bacterial population at the phylum level in the first and second groups of samples. **a** Trachea; **b** cecum

The trachea microbiota of the two groups of animals differed at the phylum level (Fig. 3a). Although Verrucomicrobia (*P* < 0.001) and Nitrospirae (*P* = 0.028) were not found in the second group, Actinobacteria (*P* < 0.001), Candidate_division_TM7 (*P* < 0.001), Acidobacteria (*P* < 0.001) and Crenarchaeota (*P* = 0.294, only 1 animal) were increased in abundance. Variation between the groups was significant with regard to Proteobacteria, Firmicutes, Fusobacteria, Planctomycetes, Chloroflexi, and Euryarchaeota (*P* < 0.001), as well as Cyanobacteria (*P* = 0.032), Chlorobi (*P* = 0.030), and Gemmatimonadetes (*P* = 0.001). Moreover, Chloroflexi, Synergistetes, Deinococcus, Deferribacteres, Lentisphaerae, Armatimonadetes and Actinobacteria were different between young and adult NMRs (*P* < 0.05). Conversely, no differences in bacterial taxa were observed between male and female NMRs (Table 2).

**Table 2:** Phyla ratio in the trachea in time, sex, and age

Phylum	First ratio				Second ratio			
	Female		Male		Female		Male	
	10~ 20 weeks	20~ 35 weeks	10~ 20 weeks	20~ 35 weeks	10~ 20 weeks	20~ 35 weeks	10~ 20 weeks	20~ 35 weeks
Proteobacteria	74.73	55.66	59.26	77.965	53.51	48.18	42.03	45.61
Firmicutes	16.03	19.73	22.66	14.865	36.18	44.98	43.62	42.87
Bacteroidetes	5.73	17.88	12.50	4.275	3.45	2.74	2.77	2.93
Fusobacteria	1.51	4.01	3.46	1.93	0.16	0.10	0.14	0.30
unclassified	1.89	2.51	2.01	0.93	4.06	0.49	3.95	5.54
Spirochaetes	0.04	0.11	0.07	0.025	0.08	0.46	0.03	0.09
Planctomycetes	0.02	0.02	0.01	0.005	0.02	0.04	0.01	0.05
Chloroflexi	0.01	0.02	0.01	/	0.02	0.05	0.03	0.05
Verrucomicrobia	0.01	0.01	0.01	/	/	/	/	/
Synergistetes	> 0.01	0.01	0.01	/	/	0.03	0.01	0.01
Cyanobacteria	0.01	0.01	> 0.01	/	/	> 0.01	> 0.01	> 0.01
Chlorobi	0.01	/	> 0.01	/	> 0.01	0.01	> 0.01	0.01
Thermotogae	0.01	/	> 0.01	/	> 0.01	> 0.01	/	/
Nitrospirae	> 0.01	0.01	> 0.01	/	/	/	/	/
Tenericutes	/	0.01	> 0.01	/	> 0.01	> 0.01	/	/
Gemmatimonadetes	> 0.01	0.01	> 0.01	/	0.01	0.01	0.02	0.01
Euryarchaeota	> 0.01	> 0.01	> 0.01	/	0.01	0.02	0.01	0.02
Fibrobacteres	> 0.01	0.01	/	0.005	0.01	0.06	> 0.01	0.04
Deinococcus-Thermus	> 0.01	/	> 0.01	0.005	/	0.01	> 0.01	> 0.01
Chlamydiae	> 0.01	> 0.01	> 0.01	/	/	> 0.01	> 0.01	> 0.01
Elusimicrobia	/	> 0.01	/	/	/	/	/	> 0.01
Deferribacteres	/	> 0.01	/	/	/	0.01	/	/
Lentisphaerae	/	> 0.01	/	/	> 0.01		/	> 0.01
Armatimonadetes	/	> 0.01	/	/	/	> 0.01	/	> 0.01
Actinobacteria	/	/	/	/	2.40	2.70	2.25	2.38
Candidate_division_TM7	/	/	/	/	0.04	0.04	0.08	0.02
Acidobacteria	/	/	/	/	0.02	0.04	0.02	0.03
Crenarchaeota	/	/	/	/	/	/	/	> 0.01

For the first group, 23 phyla were found among the cecum contents, including the highest content of Bacteroidetes (40.56%), followed by Firmicutes (26.21%), Spirochaetes (15.43%), unclassified (8.16%), Proteobacteria (4.58%), Verrucomicrobia (1.06%), and Fibrobacteres (0.74%). Although Verrucomicrobia accounted for only 1.06% of the abundance, it was the only taxon with a higher content in male young animals (10 to 20 weeks). The cecum microbiota of the second group consisted of 25 phyla, including the highest content of Firmicutes (45.81%), followed by Bacteroidetes (24.08%), Spirochaetes (23.38%), unclassified (2.61%), Proteobacteria (1.46%), Fibrobacteres (0.89%) and Actinobacteria (0.69%).

Differences in diversity at the phylum level were also found for the cecum contents between the two groups (Fig. 3b). Compared with the first group, Bacteroidetes in the second was markedly decreased (*P* < 0.001); Firmicutes (*P* = 0.026), Spirochaetes (*P* = 0.016), Tenericutes (*P* < 0.001) and Planctomycetes (*P* < 0.001) were also significantly increased and Proteobacteria (*P* < 0.001), Verrucomicrobia (*P* < 0.001) and unclassified (*P* < 0.001) significantly reduced. Deinococcus-Thermus, Actinobacteria, Acidobacteria, Candidate_division_TM7 and Chlamydiae were only present in the second group of animals, whereas Thermotogae and Thermodesulfobacteria, which were low in abundance in the first group of animals, were absent from the cecum microbiota in the second groups of animals. Additionally, Nitrospirae, Deinococcus-Thermus and Chlamydiae were rare in the second group. Regarding age, the phyla in young (10 to 20 weeks) and adult (20 to 35 weeks) animals only differed for Planctomycetes (*P* = 0.013) and Actinobacteria (*P* = 0.018); regarding gender, only Fusobacteria (*P* = 0.024) differed (Table 3).

**Table 3:** Phyla ratio in the cecum in time, sex, and age

Phylum and OTUs	First ratio				Second ratio			
	Female		Male		Female		Male	
	10~ 20 weeks	20~ 35 weeks	10~ 20 weeks	20~ 35 weeks	10~ 20 weeks	20~ 35 weeks	10~ 20 weeks	20~ 35 weeks
Bacteroidetes	38.16	47.73	40.37	35.97	21.35	22.36	20.79	32.12
Firmicutes	29.38	20.00	30.50	24.97	34.51	52.51	48.60	47.84
Spirochaetes	16.85	17.46	10.99	28.43	38.25	14.96	24.40	13.40
Unclassified	7.79	8.85	8.99	7.01	2.66	2.91	3.30	1.43
Proteobacteria	5.17	5.03	4.87	3.25	1.36	1.58	1.45	1.66
Verrucomicrobia	0.01	< 0.01	3.17	0.01	0.17	0.03	0.05	0.08
Fibrobacteres	2.01	0.51	0.45	0.02	0.08	3.19	0.16	1.53
Synergistetes	0.28	0.11	0.28	0.08	0.30	0.32	0.13	0.25
Deferribacteres	0.20	0.23	0.12	0.24	0.22	0.27	0.16	0.11
Tenericutes	0.03	0.03	0.11	0.02	0.09	0.10	0.21	0.31
Euryarchaeota	0.08	0.03	0.05	0.01	0.01	0.01	0.02	0.02
Elusimicrobia	0.03	0.02	0.05	0.01	0.04	0.01	0.03	0.01
Lentisphaerae	0.01	0.01	0.03	/	0.02	0.01	< 0.01	0.01
Chloroflexi	0.01	0.01	0.01	< 0.01	0.03	0.01	0.01	0.01
Planctomycetes	0.01	/	< 0.01	< 0.01	0.27	0.50	0.19	0.43
Gemmatimonadetes	< 0.01	/	< 0.01	< 0.01	< 0.01	/	/	/
Cyanobacteria	< 0.01	/	< 0.01	/	0.01	< 0.01	< 0.01	/
Thermotogae	/	/	< 0.01	/	/	/	/	/
Nitrospirae	< 0.01	/	/	0.01	/	/	< 0.01	/
Chlorobi	< 0.01	/	/	/	/	0.01	/	/
Fusobacteria	< 0.01	/	/	/	< 0.01	< 0.01	/	/
Thermodesulfobacteria	< 0.01	/	/	/	/	/	/	/
Deinococcus-Thermus	/	/	/	/	/	< 0.01	/	/
Actinobacteria	/	/	/	/	0.63	1.23	0.47	0.76
Acidobacteria	/	/	/	/	0.01	/	< 0.01	< 0.01
Candidate_division_TM7	/	/	/	/	< 0.01	< 0.01	< 0.01	< 0.01
Chlamydiae	/	/	/	/	< 0.01	/	/	/

### Comparison of microbiota at the genus level

**Fig. 4:**
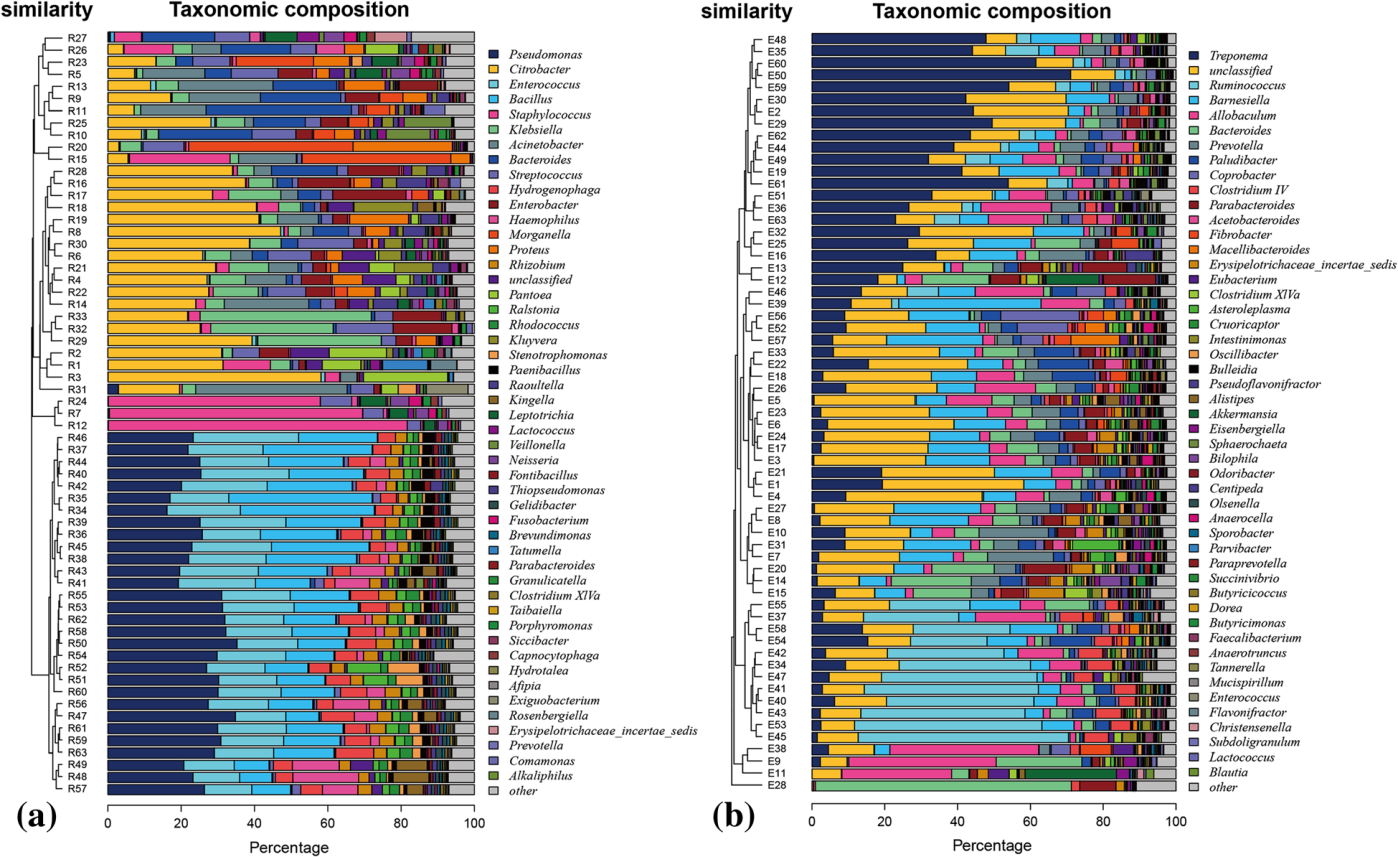
Bacterial diversity and evolutionary tree at the genus level. **a** Trachea; **b** cecum. Bacterial composition of the different communities. The relative read abundance of different bacterial phyla within the different communities. Sequences with a relative abundance lower than 1% were assigned as “others”

Based on the analysis at the genus level, the microbiota was more abundant in the trachea samples from the second group (Fig. 4a), whereas the results for the cecum (Fig. 4b) samples were the opposite.

The trachea samples from the first group comprised 533 genera, the main genus (content percentage > 1%) of which consists of 15 genera. The percentage of *Enterobacter* was the highest, followed by *Staphylococcus*, *Bacteroides*, *Klebsiella*, *Pantoea* and *Acinetobacter*. There were 733 genera in the trachea samples from the second group, the main genus (content percentage > 1%) of which had 11. *Bacillus* and *Pseudomonas* accounted for more than half of all bacteria. The next most abundant taxon was *unclassified*, followed by *Haemophilus*, *Rhizobium* and *Paenibacillus*.

Regarding the cecum samples from the first group, 385 genera were identified, the contents of which were all low. The main genus had (> 1%) 21. The percentage of *Treponema* was the highest, followed by *unclassified-Porphyromonadaceae*, *Bacteroides*, *unclassified* and *Allobaculum*. In total, 110 genera were identified in the cecum samples from the second group, the main genus (content percentage > 1%) of which had 16. On the very top of the list were *Treponema*, *Ruminococcus, unclassified-Porphyromonadaceae*, *Allobaculum* and *Inertae-Sedis*.

### NMDS (nonmetric multidimensional scaling) analysis

**Fig. 5:**
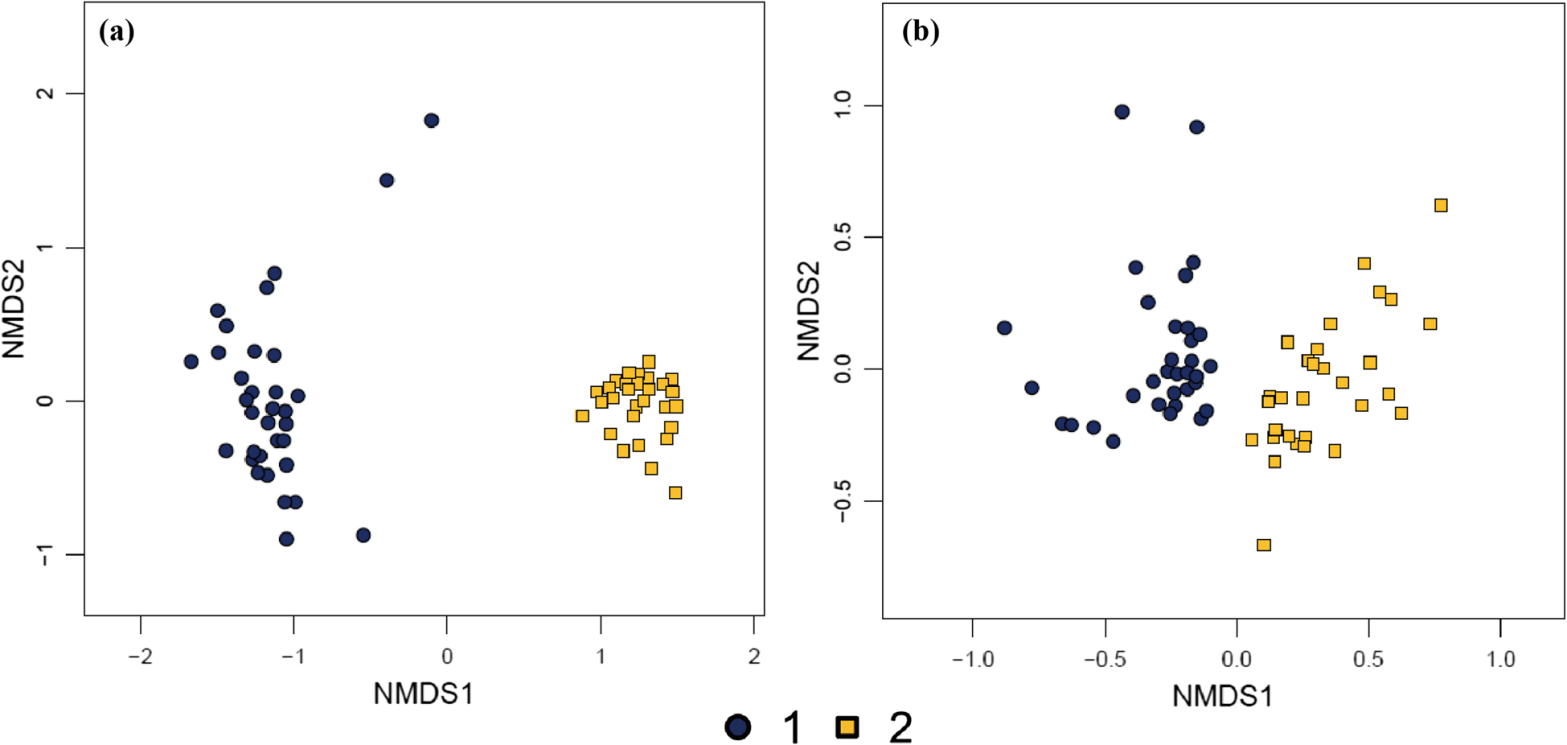
NMDS diagram of all samples. **a** Trachea samples R1-R63; **b** Cecum samples E1-E63

NMDS analysis of the trachea and cecum sequencing results (Fig. 5a, b) revealed a basically concentrated bacterial structure for the groups. Regardless of whether the samples were obtained from the respiratory or intestinal tract, the two batches of bacteria differed significantly. There were no obvious differences between females and males or young and adult animals. The findings also showed that the microbiota of the NMR trachea and intestinal tract tended to be stable after artificial feeding. However, after breeding, the microbiota in the cecum became polarized, with the microbiota in the same aged animals tending to be the same.

### Pathogens

The pathogens or conditioned pathogens identified in the trachea samples from the first group were *Klebsiella pneumoniae*, *Citrobacter freundii* [19], *Enterobacter cloacae* [20], *Proteus vulgaris*, *Proteus mirabilis*, *Staphylococcus sciuri*, *Klebsiella oxytoca*, *Aeromonas caviae*, *Streptococcus suis* and *Mycoplasma pulmonis*. The main pathogen in the cecum samples from the first group was *Klebsiella pneumoniae*.

Spirochaetaceae was always present in the cecum samples from the two groups of animals. *Mycoplasma* was present in cecum samples from the second group but not in the trachea samples, a result that may be due to residual nucleic acid.

## Discussion

For the first time, the microbiota in the trachea and cecum of NMRs in captivity was investigated using Illumina MiSeq high-throughput sequencing, and our results revealed the bacterial diversity of these two organs at the phylum and genus levels. We demonstrated that in the first group of animals, the most abundant taxon was Bacteroidetes (40.56%) in cecum samples, followed by Firmicutes (26.21%), which was similar to the result reported by Debebe [16] in which *Bacillus megaterium* accounted for 45.2% of the culture, followed by *Bacteroides thetaiotaomicron* (19.4%). We also isolated cultured bacteria from the cecum contents and performed 16S rDNA sequencing (Table 4). The main bacteria isolated from the cecum samples from the first group were Firmicutes (18 species), Bacteroidetes (10 species) and Proteobacteria (17 species); Actinobacteria and Ascomycota were the main normal microflora in the wild NMR cecum. *Klebsiella pneumonia* and *Bacillus pumilus* were isolated from the colon instead of the cecum in wild NMRs. The microflora changed in the second group of animals, which included Firmicutes (19 species), Bacteroidetes (1 species), Proteobacteria (3 species), and Actinobacteria (1 species). Notably, a significant reduction in Bacteroidetes was observed in the second group of animals, which was consistent with the result of high-throughput sequencing in which the content of Bacteroidetes was reduced by almost twofold (Fig. 2). In the same case, only 3 species of Proteobacteria were observed. The results of high-throughput sequencing showed that the level of Proteobacteria increased in the second group, which suggested that the diversity among Proteobacteria strains decreased, and that the contents of the remaining strains increased. The NMRs in the present study were different from the wild animals examined by Debebe; although the main types of bacteria were similar, the strains were significantly different. This inconsistency may be related to the transition from the wild environment to the artificial breeding environment. The findings showed that the first group of NMRs maintained the microorganisms present in the original wild state. Sequencing was used to reconstruct the changes microbiota in NMRs from wild to laboratory animals.

**Table 4:** Distribution of cultured microbiota in the cecum

Phylum	1st collection	2nd collection	Reference [16]
Firmicutes	*Allobaculum stercoricanis*	*Bacillus licheniformis*	*Bacillus megaterium*
	*Bacillus altitudinis*	*Bacillus megaterium*	*Staphylococcus gallinarum*
	*Bacillus aryabhattai*	*Bacillus pumilus*	*Staphylococcus xylosus*
	*Bacillus cereus*	*Bacillus subtilis*	*Staphylococcus sciuri*
	*Bacillus licheniformis*	*Bacillus velezensis*	*Paenibacillus spp.*
	*Bacillus megaterium*	*Brevibacillus laterosporus*	*Staphylococcus warneri*
	*Bacillus pumilus*	*Enterococcus sp.*	*Brevibacillus spp.*
	*Bacillus safensis*	*Lactobacillus animalis*	*Lysinibacillus fusiformis*
	*Bacillus subtilis*	*Lactococcus lactis*	*streptococcus mitis/oralis*
	*Eubacterium cylindroides*	*Paenibacillus amylolyticus*	*Enterococcus casseliflavus*
	*Eubacterium limosum*	*Paenibacillus glucanolyticus*	
	*Lysinibacillus sp.*	*Paenibacillus pabuli*	
	*Paenibacillus terrigena*	*Staphylococcus cohnii*	
	*Staphylococcus cohnii*	*Staphylococcus haemolyticus*	
	*Staphylococcus gallinarum*	*Staphylococcus kloosii*	
	*Staphylococcus kloosii*	*Staphylococcus pettenkoferi*	
	*Staphylococcus sciuri*	*Staphylococcus saprophyticus*	
	*Staphylococcus sp.*	*Staphylococcus warneri*	
		*Streptococcus salivarius*	
Bacteroidetes	*Virgibacillus sp.*	*Bacteroides thetaiotaomicron*	*Bacteroides ovatus*
	*Alistipes shahii*		*Bacteroides thetaiotaomicron*
	*Bacteroidaceae bacterium*		*Bacteroides fragilis*
	*Bacteroides fragilis*		*Bacteroided vulgatus*
	*Bacteroides stercorirosoris*		
	*Bacteroides thetaiotaomicron*		
	*Bacteroides xylanisolven*		
Proteobacteria	*Parabacteroides distasonis*	*Proteus penneri*	*Enterobacter cloacae/osurae*
	*Acinetobacter baumannii*	*Pseudomonas fragi*	
	*Citrobacter freundii*		
	*Enterobacter aerogenes*		
	*Enterobacter asburiae*		
	*Enterobacter cloacae*		
	*Enterobacter hormaechei*		
	*Klebsiella pneumoniae*		
	*Klebsiella variicola*		
	*Lactobacillus plantarum*		
	*Lonepinella*		
	*Morganella morganii*		
	*Pantoea agglomerans*		
	*Proteus penneri*		
	*Proteus vulgaris*		
Actinobacteria		*Microbacterium sp.*	

### The respiratory tract

Studies have shown that microbial diversity in the lung is significantly lower in patients with asthma, chronic obstructive pulmonary disease (COPD) and cystic fibrosis than in healthy individuals, mainly manifested by the increase in Proteobacteria (such as *Haemophilus*) or Firmicutes (such as *Staphylococcus*) [21]. Proteobacteria (66.90%), Firmicutes (18.32%), Bacteroidetes (10.10%) and Fusobacteria (2.73%) accounted for more than 98% of the microbiota in the trachea samples from the second group of NMRs. Analyses of human lung microbiota have revealed that there is a lower risk of later disease with great diversity in early exposure [22]. However, it is unknown whether NMRs conform to this generality. Moreover, host genes influence the upper airway microbial composition in humans and affect mucosal immunity [23]. Thus, our findings suggest that the natural anticancer properties of NMRs are largely related to their genetic properties.

Chen et al. [24] compared the microbial diversity of healthy individuals and patients with dental caries and periodontal disease and identified Firmicutes, Bacteroidetes, Proteobacteria, Actinobacteria and Fusobacteria in healthy individuals, which is similar to the microbiota observed in the NMR trachea. Interestingly, the observed microbiota changes in the trachea of NMRs in captivity (Firmicutes 18 to 42%, Proteobacteria 67 to 47%) were similar to the changes observed in oral bacteria in periodontitis patients (Firmicutes 36 to 60%, Proteobacteria 50 to 25%). Xiao et al. [25] demonstrated in rats that the microbial community in the oral cavity can be affected by an altitude-induced hypoxic environment, with more severe periodontal lesions. Therefore, it is necessary to investigate bacteria in other respiratory areas, such as the oral cavity, which may serve as a good model for oral disease studies.

The microbiota in the respiratory tract, which are more likely to be influenced by the environment, appear to not be as influential as those in the gut. Our sequencing results also showed that the bacteria in the respiratory samples from the second group of animals were significantly different from those in the first group. The number of genera increased by one-third, and the first several dominant genera were all different. This may be related to exposure to some bacteria that cannot be cleared by the body in the new environment and because microbiota of the respiratory tract were adapted to this new environment. The respiratory tract is a site of microbial entry, and these bacteria are a major factor in the maintenance of respiratory physiology and immune balance in animals [26].

The results of tracheal sequencing confirmed a certain proportion of *Pasteurella*, though the distribution bar plot figure generated by the software utilized did not list this genus (Fig. 4a); only *Haemophilus* was listed, which is likely due to the similarity in 16 s rRNA sequence [27]. *Pasteurella* is one of the most common genera in laboratory animals, and the species that have the greatest influence on laboratory animals are *P. multocida* and *P. pneumotropica*. These pathogens were not found in the NMRs in our study.

### The cecum

The results of high-throughput sequencing also confirmed that Firmicutes and Bacteroidetes are predominant bacteria in the gut microbiota. However, unlike the normal human gut flora, the abundance of Firmicutes was 3/5 that of Bacteroidetes in the first group of animals and 2/1 in the second group, which was very different from previous reports [16, 28]. This finding provides new data regarding the range of normal bacteria in the NMR.

A previous study [29] reported that changes in *Bacteroides*, *Parabacteroides*, *Alistipes* and *Akkermansia* in the gut microbiota are closely related to the occurrence of colorectal cancer (CRC). The blocking effect of the antibody targeting cytotoxic T lymphocyte antigen 4 (CTLA-4) depends on the types of *Bacteroides* in the body [13]. *Bacteroides* in NMRs in the cecum samples from the first group of accounted for 40.56% and decreased to 24.08% in the second group. These changes, including other flora, such as *Lactococcus*, can effectively inhibit the growth of cancer cells [30]. The effects of microbiota on the health and longevity of NMRs require long-term observations and further studies.

The main pathogen found in NMR was *Klebsiella pneumoniae*; moreover, it is worth noting that the highest genus content in the cecum was *Treponema* (Spirochaetaceae), which is also an important pathogen. This situation is consistent with microscopy sampling., which revealed a large number of seagull or spiral-shape long bacteria in the cecum contents. LEfSe analysis showed that Spirochaetaceae can serve as a biomarker after a period of breeding. Although the carrier rate of *Treponema* decreased after breeding, the contents of *Treponema* in most animals increased. This change may be related to the difference in breeding environment, as Debebe [17] noted that *Treponema* species increase the ability of NMRs to digest and extract valuable nutrition from fibrous naturally occurring plants, which are enriched in the NMR diet.

## Conclusions

This study laid the foundation for the laboratory animalization of this new experimental animal. The NMR microbiota composition is potentially associated with its breeding environment. The factors associated with NMR longevity may lie in the microbiota in the intestinal tract. Our results show that the microbiota of NMRs differs between the wild environment and the artificial breeding environment. The artificial breeding of NMR is intended to make better use of its antitumor and longevity properties. In captivity, changes in an animal’s microbiota may have an effect on its original characteristics, which may be the subject of further studies.

## Methods

### Sample collection

NMRs were obtained from the Laboratory Animal Center of the Second Military Medical University. The animal handling and study procedures were in accordance with the current Chinese regulation “GB14925- 2010 Laboratory animal requirements of environment and housing facilities” (Chinese version). The complete protocol was reviewed and approved by the Institutional Animal Care and Use Committee of the Second Military Medical University. The trachea and cecum were collected from 63 animals, which were sampled at random. Approximately 0.5 cm of the middle section of the trachea was removed using ophthalmic scissors and stored in sterile PBS in Corning tubes under aseptic conditions. Cecum samples were obtained by opening the abdomen and removing approximately 1 cm of the end section of the cecum, which was stored in a Corning tube. The anatomical and sampling procedures were performed in a biosafety cabinet. All samples were stored at − 80 °C and prepared for DNA extraction. Detailed information regarding the animals is provided in Table 5. In this study, NMRs between 10 and 20 weeks and between 20 and 35 weeks were defined as young and adult NMRs, respectively, for comparison of their microbiota.

**Table 5:** Data collection from NMRs

Age (week)	1st collection		2nd collection		total
	♂	♀	♂	♀	
10–15	9	7	7	6	29
16–20	5	6	2	3	16
21–25	0	2	0	0	2
26–30	2	2	8	2	14
31–35	0	0	1	1	2
Total	16	17	18	12	63

### DNA extraction and PCR amplification

Bacterial genomic DNA was extracted using E.Z.N.A. Soil DNA Kit (Omega Bio-tek, Inc., Norcross, GA, USA) according to the manufacturer’s instructions. The DNA concentration and purity were determined using a Qubit 2.0 Fluorometer (ThermoFisher Scientific, Waltham, MA, USA). The V3 and V4 hypervariable regions of the bacterial 16S rRNA were amplified using KAPA HiFi Hot Start Ready Mix (2×) (TaKaRa Bio Inc., Kusatsu, Shiga, Japan) and the following pair of 16S universal primers: 341F (5’-CCTACGGGNGGCWGCAG-3′) and 805R (5’-GACTACHVGGGTATCTAATCC-3′) [31]. The reaction was carried out as follows in a final volume of 30 μL: 2 μL of microbial DNA (10 ng/μL) as the template, 1 μL of each primer (10 μM), and 15 μL of 2× KAPA HiFi Hot Start Ready Mix. The PCR was performed using an Applied Biosystems 9700 (ABI, Foster City, CA, USA) with the following program: 95 °C for 3 min; 5 cycles at 95 °C for 30 s, 45 °C for 30 s, and 72 °C for 30 s; 20 cycles at 95 °C for 30 s, 55 °C for 30 s, and 72 °C for 30 s; and a final extension step at 72 °C for 5 min. The PCR products were evaluated by 1% (*w*/*v*) agarose gel electrophoresis using TBE buffer, staining with ethidium bromide and visualization under ultraviolet light.

### 16S gene library construction, quantification, and sequencing

After assessment, the PCR products were purified using Agencourt AMPure XP magnetic beads (Beckman Coulter, USA) to remove free primers and primer dimer species from the product. Illumina libraries were constructed using the universal Illumina adaptor and index. Prior to sequencing, the DNA concentration of each amplicon was quantified using a Qubit® 2.0 BR dsDNA assay kit (ThermoFisher Scientific, Waltham, MA, USA), and quality control was performed using an Agilent 2100 Bioanalyzer (Agilent, Santa Clara, CA, USA). Depending on the coverage needs, all libraries may be pooled for a single run. Each mixture of amplicons was pooled in equimolar ratios based on their concentration. Sequencing was performed using the Illumina MiSeq platform (Illumina MiSeq, San Diego, CA, USA) with the paired-end 300 bp at Sangon BioTech, Co., Ltd., Shanghai, China. Raw reads were deposited in the NCBI Sequence Read Archive (SRA) database under the following accession numbers: SRS2177386 and SRS2177389.

### Sequence processing and bioinformatic analysis

After sequencing, raw reads were assembled using FLASH software (v0.9.6) [32] based on overlap, and fastq files were processed to generate individual fasta and qual files. The paired-end reads in each sample were divided according to unique barcodes. Processing of the assembled sequence reads was performed using the software package MOTHUR (Version 1.30.1) [33] with the following criteria: (1) sequence lengths < 200 bp and > 480 bp were dislodged; (2) sequences containing ambiguous bases or a homopolymer greater than 6 bp in length were removed [34]; (3) bases with trailing quality scores < 20 were removed, reads with a 10-bp sliding window were scanned, and truncated reads shorter than 50 bp were discarded; (4) merged reads with a mismatch ratio in overlapping regions > 0.1 were removed; and (5) noise was removed using the Pre-cluster tool. High-quality bacterial and archaeal sequences were clustered into OTUs using Usearch (version 5.2.236) with a similarity threshold of 97% [35]. Chimeric sequences and singleton OTUs were also identified and removed using UCHIME (version 4.2.40) [36]. Taxonomy based on the 16S rRNA gene sequence was assessed using the Ribosomal Database Project (RDP) classifier [37] against the Silva database [38] at a confidence level of 0.8. Microbial diversity in the individual cecum samples was estimated using rarefaction analysis. Alpha diversity and beta diversity indexes were calculated using MOTHUR [33]. A Bray-Curtis tree of hierarchical clustering was constructed using the unweighted pair group method with arithmetic mean (UPGMA). NMDSs were all conducted using R software (version 3.2) [39]. Statistical comparisons of the microbiota composition between the first and second sequencing runs were analyzed by an independent-samples t-test using SPSS 19.0. Alpha diversity comparisons of male and female NMRs and young and adult NMRs were calculated via Kruskal-Wallis test analysis using the software LEfSe [40]. If associated sub-groups were observed, the differences between the groups and sub-groups were analyzed by a Wilcoxon rank-sum test. Finally, LDA was used to analyze the effect of these differences between the groups. The level of statistical significance was accepted as *P* < 0.05.

## Abbreviations

COPD: Chronic obstructive pulmonary disease
CRC: Colorectal cancer
FLASH: Fast length adjustment of short reads
LDA: Linear discriminant analysis
LEfSe: Linear discriminant analysis effect size
NMDS: Nonmetric multidimensional scaling
NMR: Naked mole-rat
OUTs: Operational taxonomic units
RDP: Ribosomal Database Project
SRA: Sequence read archive
UPGMA: Unweighted pair group method with arithmetic mean
